# Supplementary material for: The DNA adenine methylase of Salmonella Enteritidis promotes their intracellular replication by inhibiting arachidonic acid metabolism pathway in macrophages
Source: Front Microbiol. 2023 Mar 2;14:1080851. doi: 10.3389/fmicb.2023.1080851 (PMC10018194; doi:10.3389/fmicb.2023.1080851)
Supplement: Supplementary file 2 [file Data_Sheet_2.zip › S1 Appendix. Non-targeted metabolomics raw data/4.MetDiffAnalysis/C50336_Ddam.vs.control/ROC_neg/Com_984_neg_ROC.pdf]

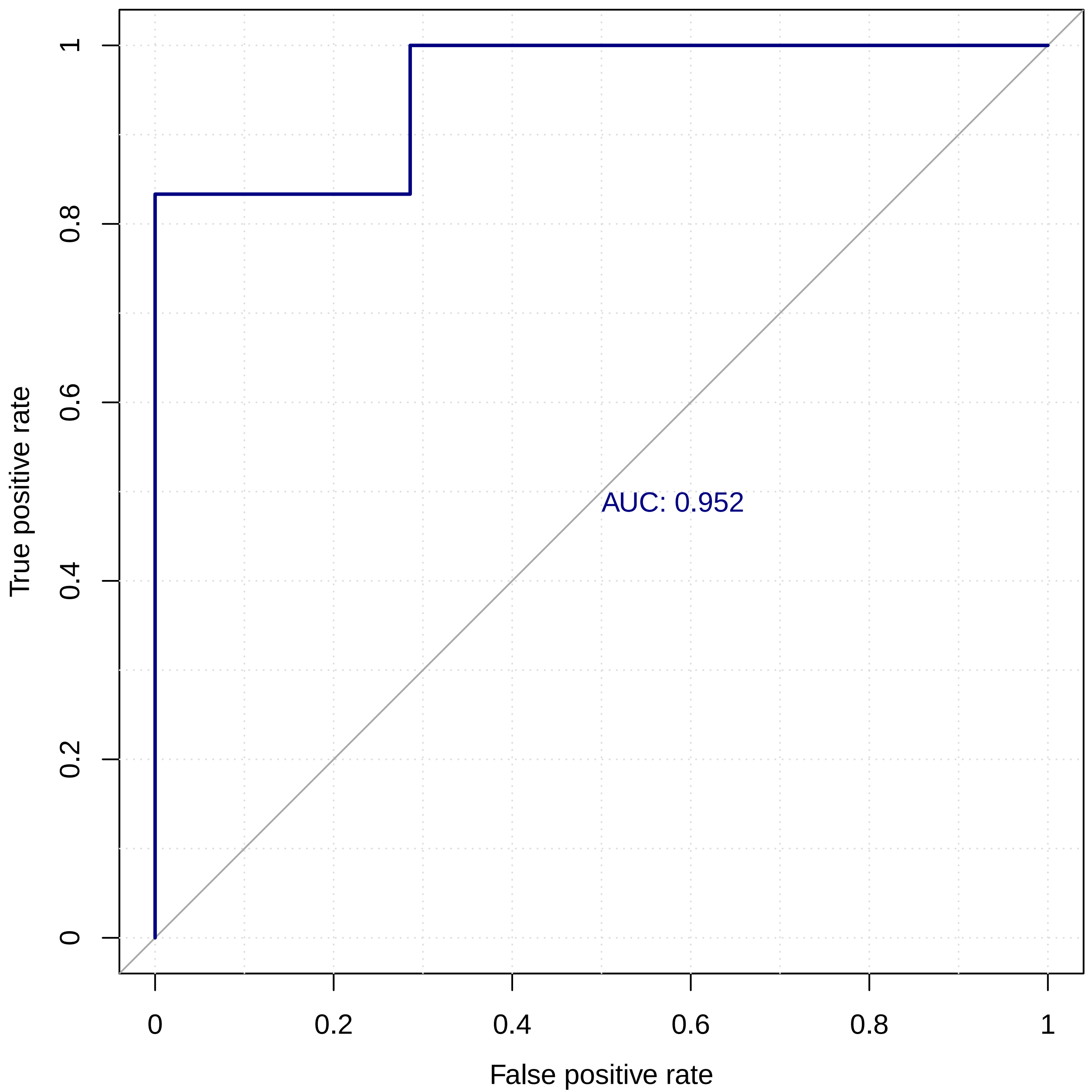

AUC: 0.952
True positive rate
0
0.2
0.4
0.6
0.8
1
False positive rate
